# Arginine Deiminase and Biotin Metabolism Signaling Pathways Play an Important Role in Human-Derived Serotype V, ST1 *Streptococcus agalactiae* Virulent Strain upon Infected Tilapia

**DOI:** 10.3390/ani10050849

**Published:** 2020-05-14

**Authors:** Yu Liu, Liping Li, Zhiping Luo, Rui Wang, Ting Huang, Wanwen Liang, Qunhong Gu, Fangzhao Yu, Ming Chen

**Affiliations:** 1 Guangxi Academy of Fishery Sciences, Nanning 530021, China; liuyudennil@163.com (Y.L.); pinglili2000@163.com (L.L.); raywongxx@163.com (R.W.); htwish@163.com (T.H.); nnlww@126.com (W.L.); 2 Zhuhai modern agriculture development center, Zhuhai 519000, China; zhluozhp@163.com (Z.L.); guqunhong2020@163.com (Q.G.)

**Keywords:** *Streptococcus agalactiae*, *Oreochromis niloticus*, cross-species infection, transcriptome, proteome

## Abstract

**Simple Summary:**

Patients who were infected with *Streptococcus agalactiae* (ST1) were mainly associated with asymptomatic carriage. However, the invasive diseases in non-pregnant adults caused by *S. agalactiae* (serotype V, ST1) have increased recently. We have previously reported that human-derived *S. agalactiae* (serotype V, ST1) could infect tilapia with virulence and pathologic characteristics similar to highly virulent tilapia-derived *S. agalactiae* (ST7) strains. The potential risk of cross-species infection cannot be ignored. Therefore, our research provided a multi-omics analysis of the human-derived serotype V ST1 *S. agalactiae* strains, which were virulent and non-virulent to tilapia and provided a more comprehensive understanding of the virulence mechanism.

**Abstract:**

Our previous study showed that human-derived *Streptococcus agalactiae* (serotype V) could infect tilapia, but the mechanism underlying the cross-species infection remains unrecognized. In this study, a multi-omics analysis was performed on human-derived *S. agalactiae* strain NNA048 (virulent to tilapia, serotype V, ST1) and human-derived *S. agalactiae* strain NNA038 (non-virulent to tilapia, serotype V, ST1). The results showed that 907 genes (504 up/403 down) and 89 proteins (51 up/38 down) were differentially expressed (*p* < 0.05) between NNA038 and NNA048. Among them, 56 genes (proteins) were altered with similar trends at both mRNA and protein levels. Functional annotation of them showed that the main differences were enriched in the arginine deiminase system signaling pathway and biotin metabolism signaling pathway: gdhA, glnA, ASL, ADI, OTC, arcC, FabF, FabG, FabZ, BioB and BirA genes may have been important factors leading to the pathogenicity differences between NNA038 and NNA048. We aimed to provide a comprehensive analysis of the human-derived serotype V ST1 *S. agalactiae* strains, which were virulent and non-virulent to tilapia, and provide a more comprehensive understanding of the virulence mechanism.

## 1. Introduction

*Streptococcus agalactiae* not only causes pneumonia and meningitis in humans, but also causes streptococcosis in farmed tilapia [1,2,3]. However, some zoonotic/multi-host lineages have emerged recently, increasing the risk of foodborne and zoonotic infections [4,5,6]. Patients who were infected with *S. agalactiae* (ST1) were mainly associated with asymptomatic carriage, but the invasive diseases in non-pregnant adults caused by *S. agalactiae* (serotype V, ST1) have increased recently [7,8,9,10]. We have previously reported that human-derived serotype V, ST1 *S. agalactiae* could infect tilapia, [5] which has similar virulence and pathologic characteristics to highly virulent tilapia-derived *S. agalactiae* (ST7) strains [11]. The potential risk of cross-host infection cannot be ignored.

Studies on human-derived *S. agalactiae* using molecular biology techniques have identified proteins associated with the virulence of *S. agalactiae,* including sialic acid-rich capsular polysaccharides [12], fibronectin-binding proteins [13], pili [14], serine rich chromatin repeat protein [15], fibrinogen-binding protein [16], hyaluronan lyase [17], β-hemolysin/cytolysin [18], cAMP factor [19], complement C5a peptidase [20], and superoxide dismutase [21]. With the development of sequencing technologies, gene annotation of fish-derived *S. agalactiae* virulent strains has gradually been completed in whole or in part [22,23,24,25]. Genome-wide studies are beginning to gain functional applications in the control and prevention of diseases in teleosts fish [26]. When compared with human-derived genomes, the key virulence factors of fish-derived *S. agalactiae* were different. However, different serotypes of *S. agalactiae* from the same host have different key virulence factors [25,26,27,28]. Therefore, the virulence mechanism of the cross-species infection of human-derived serotype V, ST1 *S. agalactiae* may be quite different from the previous studies, and needs further investigation.

Previous virulence tests showed that human-derived *S. agalactiae* NNA048 (serotype V, ST1) was virulent to tilapia (LD_50_ = 2.66 × 10^5^ cfu/fish), while human-derived *S. agalactiae* NNA038 was completely non-pathogenic to tilapia [5]. A histopathological section showed NNA048 caused degeneration and necrosis in various tissues, while no lesions were found in tse tilapia being challenged with NNA038. The main differences between NNA048 and NN1038 in the genome were the phage sequences: NNA048 specificcally possesses an intact phage sequence which encodes 68 proteins [11]. Based on this, a multi-omics analysis was performed to investigate the differences in mRNA and protein levels between NNA048 and NNA038, with the aim to provide a more comprehensive understanding of the virulence mechanism.

## 2. Methods

### 2.1. Bacterial Strains

The *S. agalactiae* strains NNA038 (serotype V, ST1) and NNA048 (serotype V, ST1) were isolated from two female patients with typical clinical and pathogenic characteristics of a premature rupture of fetal membranes in 2014 (Guangxi, China). The stored strains were removed from a −80 °C environment, and cultured on the blood agar plate at 30 °C for 24 h. Then, a single colony was picked up and inoculated into 10 mL of TSB medium, shaken and cultivated at 30 °C for 12 h, repeated twice. The bacterial density was calculated (CFU mL^−1^) by the flat colony counting method.

The healthy experimental tilapia were provided by National Tilapia Seed Farm (Nanning, Guangxi, China), and were similar in shape, with average weight of 400 ± 15.10 g. The fish were monitored in a sterile plastic tank with a recirculation system and biofilters for one month before the virulence test. During this period, we randomly selected one fish/day for bacteriological analysis. All fish were negative for *S. agalactiae* infection.

### 2.2. Virulence Test

A total of 36 fish were randomly selected from the tank. Among them, 12 fish were treated as a virulent group, which were challenged with NNA048 (10^9^ CFU/fish). Twelve fish were treated as an avirulent group, which were challenged with NNA038 (10^9^ CFU/fish). Twelve fish were treated as a blank control group, which were challenged with an equivalent amount of sterile PBS. Briefly, the fish were anesthetized with 10 mg L^−1^ of MS-222 (Sigma-Aldrich), and when the fish showed no stress response they were challenged with NNA048, NNA038 or PBS, respectively. Then, the three groups were monitored in individual tanks. When the challenge groups appeared dead, all the experimental fishes were executed, and the brain tissues were sampled for bacteriological analysis.

Animal experiments were conducted in strict accordance with the “Chinese animal experiment ethical inspection”, under project licence number: GXU2015039, and approved by the Guangxi University, CHINA.

### 2.3. RNA-Sequencing

Refer to our previous study [29], the cultured strains were stored in TRizol and transported with liquid nitrogen. RNA sequencing was performed by Novogene Co, LTD (Beijing, China). The total RNA was extracted by RNAiso Plus (Takara Bio, Beijing, China). After detection of the purity, concentration and integrality, 3 µg of each RNA sample was used for sequencing library generation, with the NEBNext^®^ Ultra™ Directional RNA Library Prep Kit for Illumina^®^ (NEB, Ipswich, MA, USA) [30]. Briefly, the mRNA was obtained by removing the rRNA. Then, the mRNA was fragmented and used as a template to generate the double-strand cDNA (random hexamer primer, M-MuLV Reverse Transcriptase, DNA Polymerase I and RNase H). Special adaptors were added to the fragments, which were then purified with the AMPure XP system. The selected cDNA fragments were amplified by PCR. The final library was obtained after purification and quality checking.

### 2.4. Data Analysis of the Transcripts

After removing the unqualified reads from the raw data, clean reads were obtained and mapped to the reference genome (NCBI, Accession NO.NZ_CP011325.1) by using STAR (v2.5.1b). The reads numbers of each gene were calculated by HTSeq v0.6.1 [31], and measured with FPKM numbers [32]. DESeq R package (1.18.0) provided a differential expression analysis of the two groups, and genes with an adjusted *p*-value < 0.05 were assigned as differentially expressed (adjusted with Benjamini and Hochberg’s approach) [33]. The statistical DEGs were annotated and enriched by Gene Ontology and KEGG databases, which were performed by GOseq [34] and KOBAS v2.0 [35], respectively.

The raw data’s accession numbers were <SRR7829616> and <SRR7830599> for NNA038 and NNA048, respectively (NCBI).

### 2.5. Protein Extraction and Peptide Preparation

The strain samples were lysed in the glass homogenizer individually with 1 mL of lysis buffer (50mM Tris buffer, 8M Urea, 1%SDS, pH = 8). The homogenate was disrupted by ultrasonic, then centrifuged at 12,000 g for 15 min at 4 °C. The supernatant was mixed with four volumes of 10 mM DTT in cold acetone (10%), after determining the protein concentration (Bradford assay). Static settled at −20 °C for 2 h. The sediments were collected after centrifugation, washed twice with cold acetone and dissolved in the buffer (50 mM Tris buffer, 8 M Urea, pH = 8).

After trypsin digestion, the peptide was desalted with a C18 cartridge to remove the high urea, and the desalted peptides were dried by vacuum centrifugation.

### 2.6. iTRAQ Labeling, HPLC Fractionation and LC-MS/MS Analysis

The desalted peptides were labeled with iTRAQ reagents by iTRAQ^®^ Reagent-8PLEX Multiplex Kit (Sigma). Differently labeled peptides were mixed as one sample, and desalted in 100 mg SCX columns. The desalted mix was fractionated by a C18 column on a Rigol L3000 HPLC operating at 1ml/min. The gradient elution column consisted of Mobile phases A (2% acetonitrile, 20 mM NH_4_FA, pH = 10.0) and B (98% acetonitrile, 20 mM NH_4_FA, pH = 10.0) and set as: 3%–8% B, 5 min; 8%–18% B, 12 min; 18%–32% B, 11 min; 32%–45% B, 7 min; 45%–80% B, 3 min; 80% B, 5 min; 80%–5%, 0.1 min, 5% B, 7 min. The eluent was collected once per minute and merged to 15 fractions, then dried under a vacuum and reconstituted in acetonitrile water for LC-MS/MS analyses (Easy nanoLC 1200, Q Exactive HF-X).

### 2.7. The Identification and Quantitation of Protein

Proteome Discoverer2.2 software was used to search the resulting spectra of each fraction from the “Run2_*Streptococcus_agalactiae*_GCF_001190805.1_ASM119080v1_protein.fasta” database (mass tolerance: precursor ion scans, 10 ppm; product ion scans, 0.02 Da). Carbamidomethyl was used as the fixed modification, while oxidation of methionine, acetylation of the N-terminus and iTRAQ 8-plex of tyrosine and lysine were used as variable modifications (miscleavage sites ≤ 2).

Reporter Quantification (iTRAQ 8-plex) was used for iTRAQ quantification. The protein quantitation results with *p* < 0.05 and |log2FC| >1.2 (ratio > 1.2 or ratio < 0.83 (fold change, FC)) were defined as differentially expressed proteins (DEP) (Mann–Whitney Test).

The raw data of mass spectrometry used dataset identifier < PXD011206> (ProteomeXchange Datasets).

### 2.8. Data Analysis of Proteome

InterProScan 5 was used to classify the functions of the DEPs by searching the GO, InterPro, COG and KEGG databases [36]. STRING-db was used to predict the interactions of the proteins [37]. The enrichments were performed by pipelines [38].

### 2.9. Real-Time Quantitative PCR (RT-qPCR)

The target genes were predicted to be associated with virulence. RT-qPCR was used to compare the expression levels of the target genes between NNA038 and NNA048. Primer Premier (Ver. 5.0) was used to design the specific primers of the target genes, and recA [39] gene was used as the reference gene. The total RNA was extracted from the culture strains by RNAiso Plus (Takara Bio, Beijing), then the reverse transcription and Real-time qPCR were performed by PrimeScript™ RT reagent Kit (Takara Bio, Beijing, China). The expression level was determined as a relative expression to recA using the 2(-ΔΔC(T)) method.

## 3. Results

### 3.1. Virulence Test

The results showed that NNA048 was virulent and NNA038 was avirulent to tilapia, which was consistent with our previous studies [11].

### 3.2. Statistic Analysis of the Multi-Omics Profiles

**Figure 1:**
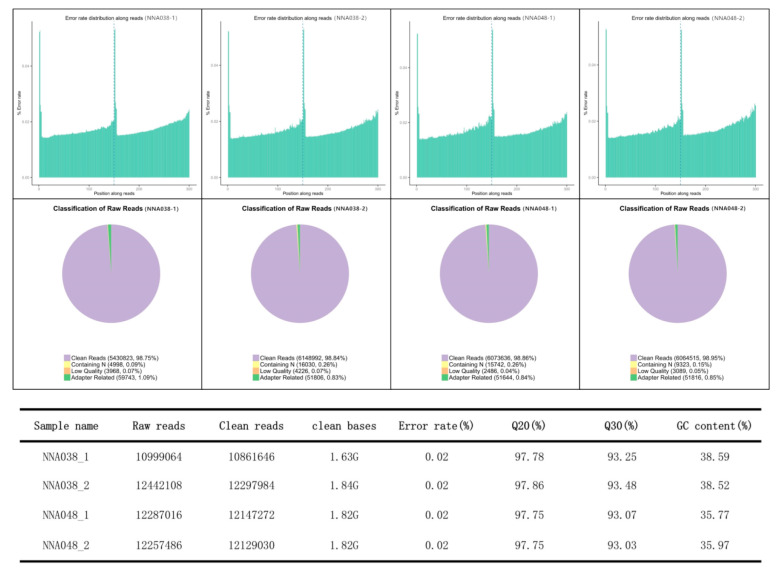
Statistics of the transcript profiles. The qualification rate of the RNA-seq is up to standard.

**Figure 2:**
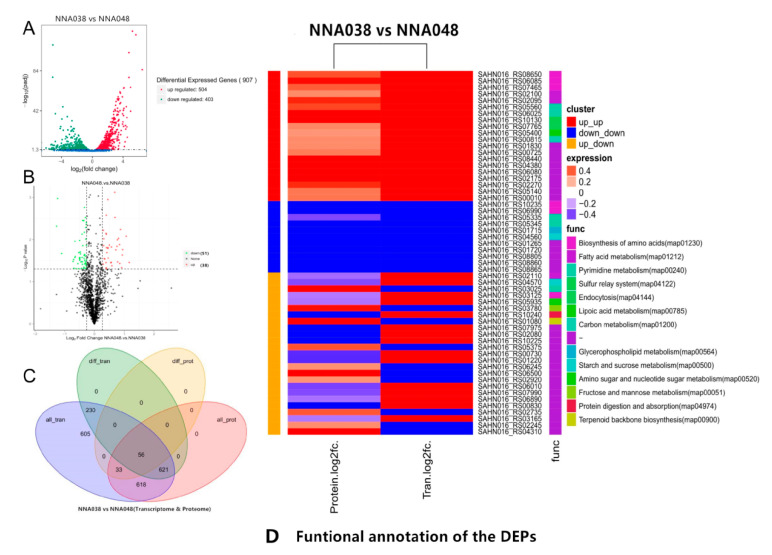
Differential expression genes and proteins analysis. (**A**). Transcriptomic analysis of differentially expressed genes in NNA038 and NNA048. (**B**). Proteomic analyses of differentially expressed proteins in NNA048 and NNA038. (**C**). Venn diagram of the DEGs and DEPs. (**D**). Functional annotation of the DEPs.

In RNA-seq profiles, a total of 47,985,674 raw reads (10,999,064 and 12,442,108 for NNA038 as well as 12,287,016 and 12,257,486 for NNA048, respectively) were generated. After removing the unqualified reads, 47,435,932 clean reads were obtained. Approximately 92.15% (89.61%–96.70%) of the mapped reads were acquired from the RNA-seq experiment, of which 90.76% (86.88%–95.45%) were mapped to unique genomic locations (Figure 1). A total of 907 genes (504 up/403 down) (Figure 2A) and 89 proteins (51 up/38 down) (Figure 2B) were significantly altered (*p* < 0.05) in the NNA038 vs NNA048 group. Among them, 56 proteins were significantly altered with similar trends at both mRNA and protein levels (Figure 2C). Functional annotation of them showed that they were mainly enriched in the biosynthesis of amino acids, fatty acid metabolism and pyrimidine metabolism pathways (Figure 2D).

### 3.3. Functional Annotation of DEGs and DEPs

The differentially expressed genes and proteins between NNA038 and NNA048 were annotated and enriched by GO and KEGG databases. By GO analysis, DEGs were mainly enriched in the organonitrogen compound metabolic process, the small molecule metabolic process and the organonitrogen compound metabolic process. The DEPs were mainly enriched in nitrogen compound transport, isomerase activity and catalytic activity. By KEGG pathway analysis, the DEGs were mainly enriched in oxidative phosphorylation, a biosynthesis of antibiotics and fatty acid metabolism. The DEPs were mainly enriched in a biosynthesis of amino acids, fructose and mannose metabolism, arginine biosynthesis and biotin metabolism.

**Figure 3:**
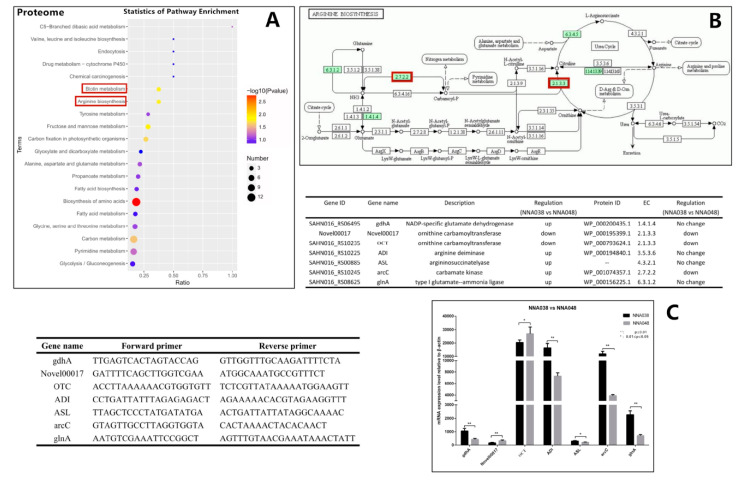
Arginine biosynthesis signal pathway and RT-qPCR verification. (**A**) showed the enrichment results of the differentially expressed proteins (DEPs). (**B**) showed the significant enriched pathway, arginine biosynthesis signaling pathway. (**C**) showed the verification result by RT-qPCR. The relative mRNA expression levels of the related genes were validated by RT-qPCR, NNA038 and NNA048 reached the significant level of * 0.01≤ *p* ≤ 0.05. ** indicates that the difference in gene expression between NNA038 and NNA048 reached the significant level of *p* ≤ 0.01.

Among the enriched signaling pathways, the arginine biosynthesis and biotin metabolism pathways were significantly enriched, and considered to be an important reason for the significant difference between NNA038 and NNA048 in the pathogenicity of tilapia (Figure 3A).

### 3.4. RT-qPCR

**Figure 4:**
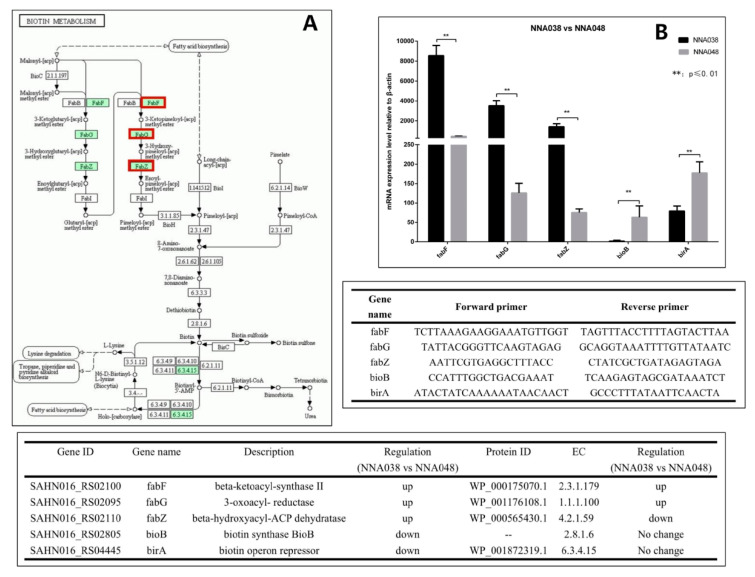
(**A**) Biotin metabolism signaling pathway and RT-qPCR verification. The relative mRNA expression levels of the related genes were validated by RT-qPCR. (**B**) NNA038 and NNA048 reached the significant level of 0.01≤ *p* ≤ 0.05. ** indicates that the difference in gene expression between NNA038 and NNA048 reached the significant level of *p* ≤ 0.01.

RT-qPCR was used to verify the expression levels of the target genes between NNA038 and NNA048. The target genes in arginine biosynthesis and biotin metabolism signaling pathways were verified in Figure 3 and Figure 4, respectively. The relative expression of the target genes were significantly different between NNA038 and NNA048, which has similar alteration trends to the multi-omics results.

## 4. Discussion

As a multi-host infectious pathogen, the risk of cross-host infection of GBS has been widely concerning [5,40,41,42,43,44,45]. It had been reported that human-derived *S. agalactiae* (ST23, ST7, ST19 and ST17) could infect fish, causing invasive disease and even death [4,27,28,46,47]. In addition, the consumption of raw fish has caused invasive streptococcosis in humans [6]. The streptococcosis could outbreak in a short time by spreading through water and densely farmed fish. At present, the treatment for streptococcosis in aquaculture is mainly dependent on antibiotics. However, this causes water pollution and resistant strains, which leads to a vicious circle. The cross-species infection between humans and fish poses a serious threat to human health. Hetron et al. have provided an overview of the progress and prospects of functional genomics based on conventional single gene sequencing, cloning and characterization together with HTS in the prevention and control of fish diseases in aquaculture, which showed that genomics studies made a great contribution in the prevention and control of fish diseases in aquaculture [26]. It has been reported that the proportion of infection caused by serotype V, ST1 *S. agalactiae* in non-pregnant adults has increased, which may cause infant death [7,8,9,10,48,49]. Therefore, the highly virulent strains which could spread across species need to be a higher concern. Human-derived *S. agalactiae* NNA048 and NNA038 (Serotype V, ST1) could both infect tilapia, but the pathogenicity was quite different. Genome study showed that there were major differences in phage sequences, NNA048 specificcally possesses an intact phage sequence which is encoded with 68 proteins [11]. In this study, 56 proteins were significantly differentially expressed between NNA048 and NNA038 with similar alteration trends at both mRNA and protein levels by multi-omics analysis. These DEGs and DEPs may be closely associated with the pathogenicity of the strains. Functional analysis of them will provide a comprehensive understanding of the virulence mechanism.

The gastrointestinal tract is the main route for a *S. agalactiae* infection. When a pathogen invades the host through the gastrointestinal tract, it must first adapt to the acidic conditions and survive in the stomach to start its infectious cycle [50,51]. Acid resistance is an important factor affecting bacterial pathogenicity. The arginine deiminase system (ADS) of the bacteria allows the bacteria to survive potentially lethal acidification through production of ammonia to raise the environmental pH value [51]. Curran et al. found that the ADS in mutans streptococci could protect it against acid damage [52]. Xiong et al. also found that ADS could help *Laribacter hongkongensis* survive in the hostile acidic environment of the stomach [53]. ADS consists of three enzymes: arginine deiminase, ornithine carbamoyltransferase and carbamate kinase [52]. The ADS-encoded genes are commonly organized as an operon, a lot of genes have been found associated with the ADS functions, but the arrangement and components differ among species [54,55,56,57,58]. In this study, we have identified some over-expressed genes which are homologous to the published *S. agalactiae* ADS-encoding genes. These genes were predicted to participate in ADS functions. The results showed that the ASL, ADI, gdhA, glnA and arcC genes were significantly down-regulated in NNA048, while OTC encoding genes were up-regulated in NNA048, which leads to ornithine carbamoyltransferase and carbamate kinase having significantly higher expression in NNA048. Therefore, NNA048 could provide more ATP for growth and proliferation derived from catabolism of arginine to ornithine, CO_2_, and NH_3_. In addition, the high concentration of ammonia is toxic to most fish because exposure of the brain to elevated ammonia concentrations leads to a wide range of neuro-cognitive deficits, intellectual disabilities, coma and death [49], which are the typical symptoms of GBS diseases [59]. The differences in ADS pathways may be the key cause of the difference between the pathogenicity of NNA038 and NNA048.

The synthesis of fatty acids is necessary because the bacterial fatty acids are important cellular components, such as the cell membrane [60]. Fatty acid biosynthesis in bacteria is carried out by the type II fatty acid synthase (FAS) system, which consists of four basic reactions. Each of the reactions is performed by discrete enzymes, which are highly specific to different species of bacteria [61]. We have shown that FabF, FabG and FabZ homologous genes in fatty acid metabolic signaling pathways are differentially expressed in NNA038 and NNA048. The enzymes encoded by these genes catalyze one of the reactions in the type II FAS system [62,63,64]. The differential expression of these coding genes leads to significant down-regulation of FabF and FabG proteins, and significant up-regulation of FabZ protein in NNA048. These changes affect the downstream biotin metabolic signaling pathway because the biotin synthase BioB and biotin operon repressor BirA genes are significantly down-regulated in NNA038, which affects the synthesis of biotin [65,66]. Biotin, a water-soluble vitamin of the B complex, functions as a cofactor of carboxylases that catalyzes an indispensable cellular metabolism. Studies have shown that biotin deficiency up-regulates TNF-alpha production in murine macrophages, which affects the cytoactive of macrophages [67]. It has been shown that GBS can survive for a long time after being phagocytosed by phagocytic cells such as macrophages and neutrophils [67,68,69], thereby escaping the killing effect of active antibacterial molecules in the blood [12,50,69]. Therefore, the differences in fatty acid metabolic pathways lead to differences in the growth and proliferation abilities of NNA038 and NNA048, which ultimately leads to differences in pathogenicity to tilapia. FabF, FabG, FabZ, BioB and BirA genes might be the important genomics-driven targets for antibacterial drug discovery.

## 5. Conclusions

In this study, a multi-omics analysis was performed on a highly virulent human-derived *S. agalactiae* strain NNA048 (serotype V, ST1) and avirulent strain NNA038 (serotype V, ST1). We have elucidated the differences between the two strains at both the mRNA and protein levels. The differences in fatty acid metabolic pathways (FabF, FabG, FabZ, BioB and BirA) and the arginine deiminase system (gdhA, glnA, ASL, ADI, OTC and arcC) may lead to the differences in pathogenicity to tilapia among the two strains. This study provided a more comprehensive understanding of the virulence mechanism of the human-derived serotype V, ST1 *S. agalactiae* strains, and some potential targets for antibacterial drug discovery were identified.
